# Socioeconomic Inequalities in Times of COVID-19 Lockdown: Prevalence and Related-Differences in Measures of Anxiety and Stress in Palestine

**DOI:** 10.3389/fpsyg.2022.898845

**Published:** 2022-06-14

**Authors:** Hamzeh Al Zabadi, Maryam Haj-Yahya, Noor Yaseen, Thair Alhroub

**Affiliations:** ^1^Department of Public Health, Faculty of Medicine and Health Sciences, An-Najah National University, Nablus, Palestine; ^2^Department of Medicine, Faculty of Medicine and Health Sciences, An-Najah National University, Nablus, Palestine

**Keywords:** anxiety, COVID-19, lockdown, quarantine, stress

## Abstract

**Background:**

Implementation of quarantine and lockdown to COVID-19 pandemic has created dramatic negative psychological impact mainly the general population’s health worldwide. We aimed to assess the prevalence and predictors of anxiety and stress severity among the Palestinian population.

**Methods:**

A cross-sectional web-based survey was conducted. An anonymous online questionnaire and snowball recruiting technique were used to target the general public in Palestine between 6 and 16 April, 2020 during COVID-19 pandemic lockdowns. Multivariate logistic regression models were developed for the outcome variables.

**Results:**

Of the 2819 individuals who completed the questionnaire, more than two thirds of them (72.6%) were females. Nearly (83.5%), were residing at the West Bank. The mean age of participants was 29.47 (*SD* = 10.97) years. The anxiety prevalence was (25.15%) with (20.08%) had mild/moderate severity. The stress prevalence was (38.77%) with (22.21%) had mild/moderate severity. The prevalence of both anxiety and stress was (20.3%). In multivariate analysis, exposure to confirmed case of COVID-19, inadequacy of food supply and jobs that acquire leaving home during lockdown were significantly related to higher anxiety degree. As for stress, low monthly income, cohabitation with a person of a high-risk group and inadequacy of food supply were significantly related to higher stress degree.

**Conclusion:**

Young adults with low socioeconomic status and inadequate food supply were more likely to have a higher degree of stress and/or anxiety. Providing alternative economical sources for those in need, and spreading more awareness regarding the pandemic, supporting the population’s psychological wellbeing, community connection and the availability of specialist mental health services are crucial to overcome the mental impacts of COVID-19 in Palestine.

## Background

On 31st December 2019, several cases of pneumonia with an unknown etiology were identified in Wuhan, China (Lu et al., 2020). By January 30th, 2020, the World Health Organization (WHO) declared the COVID-19 outbreak in China as a public health emergency of international concern casting a threat over countries with vulnerable health systems (Amer, 2018). In reaction to that, a lockdown was implemented during the COVID-19 pandemic, nearly at the global level (Brooks et al., 2020).

During outbreaks of infection, there would be a wide range of negative psychosocial impact on people as past studies shown (Hall et al., 2008). The ongoing COVID-19 pandemic is prompting fear, as people are likely to experience fear of getting infected or dying, feelings of helplessness, and stigma (Hall et al., 2008; Rubin et al., 2010; Wang et al., 2020). Recent studies of the impact of COVID-19 pandemic on mental health showed that post-traumatic stress disorder (PTSD) rates reaching up to 41% since the outbreak. Multiple factors associated with increasing psychological disturbances were identified, including female sex, lower socioeconomic status, interpersonal conflicts, frequent social media use and lower resilience and social support (Torales et al., 2020). The dynamics of psychological disturbances and their emergence during a pandemic is interwoven with daily activities. People would always seek information and updated data on the ongoing crisis, therefore, lacking of possible entrusted official resources for such information can drive people to the doubted, less accurate, fearful and highly exaggerated (European Observatory on Health Systems Policies, 2022). Social media windows usually have plenty of falsehoods, misinterpreted numbers and headlines, all together coming to burden the public leading to a fertile environment. In this situation, vulnerable populations, those of high risk for infection, being with a pre-existing medical condition, those in contact with confirmed cases of COVID-19 such as health care providers and the families of COVID-19 patients, can suffer far more than the general public during the pandemic (European Observatory on Health Systems Policies, 2022).

A meta-analysis with 71 studies showed that healthcare workers are experiencing significant levels of anxiety during the COVID-19 pandemic. The pooled prevalence of anxiety in healthcare workers including nurses, medical doctors and frontline healthcare workers was estimated to be 25% (Santabárbara et al., 2021a).

The implications of lockdown as a global response to COVID-19 may create a dramatic psychological and emotional impact on people (Hawryluck et al., 2004). According to updated meta-analysis of community-based studies during the COVID-19 pandemic, anxiety in the general population has increased threefold, and it appears to be highest at the initial phase and at the peak of the waves, with a prevalence of 25% as an average of 43 large studies (Santabárbara et al., 2021b). Mandatory contact tracing and 14 days quarantine could increase patients’ and contacts’ anxiety and guilt about the association of contagion, quarantine, and stigma on their families and friends (Xiang et al., 2020). Those in quarantine might experience boredom, loneliness, and anger. Isolation from loved ones, the loss of freedom, and the closure of schools and business, negative emotions experienced by individuals are compounded (Van Bortel et al., 2016).

Moreover, a Systematic review concluded that sleep disturbances are among the psychological outcomes that affect general population during tough times of pandemic, and can cause depression and anxiety (Alimoradi et al., 2021). Another meta-analysis study that included 16 previous studies reported an average prevalence of anxiety of 38.12% among the general population (Necho et al., 2021). A Canadian study reported 28.9% post-traumatic stress disorder (PTSD) during the SARS outbreak. According to the same study, longer durations of quarantine were associated with an increased prevalence of PTSD symptoms. Acquaintance with or direct exposure to someone with a diagnosis of SARS was also associated with PTSD and depressive symptoms (Hawryluck et al., 2004). Many of the investigations on the psychological impact on the non-infected community, revealed significant psychiatric morbidities which were found to be associated with younger age and increased self-blame. Those who were older, female gender, more highly educated, with higher risk perceptions of SARS, a moderate anxiety level, a positive contact history, and those with SARS-like symptoms were more likely to take precautionary measures against the infection (Wang et al., 2020).

Surprisingly, positive aspects of the lockdown were identified by participants in a cross-sectional study in New Zealand for themselves personally and/or for society, such as more family time, work flexibility, social cohesion, the recreation of healthy habits, considering priorities in life, and environmental benefits brought by reduced travel (Every-Palmer et al., 2020).

On 22nd March 2020, Palestine went into a lockdown, requiring social distancing, wearing masks, postponing social events or even canceling them, closing markets, restaurants, gyms, banks, schools, universities, etc. That had been last 43 days (up to 5th May 2020). The psychological and coping responses of the community during the lockdown are unknown. This study aimed to assess the prevalence and predictors of anxiety and stress severity among the Palestinian population. Understanding the experiences of the population during lockdown is critical to maximize infectious disease containment and minimize the negative associations on those quarantined, their families, and social networks.

## Materials and Methods

### Study Population, Sample, and Setting

The population was comprised of all people 18 years or older living in Palestine during the lockdown. We adopted a cross-sectional survey design to find the prevalence of stress and anxiety among the public and to identify the possible risk factors during the COVID-19 pandemic by using an anonymous online questionnaire. A snowball sampling strategy focused on recruiting the general public. The online survey was first disseminated as link to the Google form on Facebook to friends and they were encouraged to pass it on to others. Facebook was the best available option in Palestine during the lockdown.

As our sampled population was larger than 20000 (2.5 million above 18 years old), and with an expected prevalence proportion in the population between (0.2–0.5), a sample size between (246–385) or more was calculated as the minimum number of necessary subjects to meet a confidence level of 95% that the real value will be within ±5% margin of error of the measured/surveyed value with a power analysis of 20% according to the equation of unlimited population:

***n*** **=Z^2^ x P (1-P)** /ε^2^; where z is the z score; p the population proportion; ε the margin of error and n the sample size.

### Procedure

As the Palestinian Government recommended the public to minimize face-to-face interaction and individual isolation at home, potential respondents were electronically invited. They completed the questionnaires in Arabic through an online Google Form survey. Expedited ethics approval was obtained from the Institutional Review Board (IRB) at An-Najah National University (Faculty of Medicine and Health Sciences). Privacy and confidentiality of personal data were strictly protected during the procedure. The aims and the information about the study including objectivity, beneficence, non-maleficence, individual autonomy, and justice (fairness) were posted on the first page of the questionnaire. All respondents provided electronic informed consent before starting the questionnaire. The IRB approved our request for a waiver of documentation of this method of obtaining consent. All collected data transferred automatically to a protected excel sheet and only the researchers had access to this information. Data collection took place over 10 days (6–16 April 2020), 2 weeks after the beginning of the COVID-19 lockdown. At that time, lockdown and strict measures of social distancing and isolation were applied by the force of law.

### Survey Development

It is worth mentioning that this study was published as a preprint in the research square platform (Al Zabadi et al., 2021a). Previous surveys on the assessment of mental health during the lockdown during outbreaks were reviewed (Liu et al., 2020). The authors included additional questions related to the COVID-19 outbreak in Palestine. The structured questionnaire consisted of questions that covered several areas: (1) informed consent, (2) socio-demographic characteristics, (3) knowledge and concerns about the lockdown, (4) precautionary measures against COVID-19, and (5) the Depression Anxiety Stress Scales (DASS). All of these sections, except the DASS scale section, were developed by the authors. A pilot study was performed on a small group of volunteers for feedback to identify ambiguities, difficult questions, record the time needed to complete the online questionnaire; thus, minor rewording was done to clarify the meaning and questions related to the COVID-19 pandemic and lockdown. This study is a continuation of a two published papers regarding depression (Al Zabadi et al., 2020) and quarantine understanding and adherence (Al Zabadi et al., 2021b) on the same population.

### Study Measures

In the socio-demographic characteristics section, respondents were asked to answer questions about their age, sex, educational level, social status. Information also included self-report of cohabitation with someone who is of a high-risk group (pre-existing medical condition, those of high risk for infection, those in contact with confirmed cases of COVID-19 such as health care providers and the families of COVID-19 patients). Furthermore, according to the last update of the Palestinian Central Bureau of Statistics (PCBS) in 2017, the average monthly household expenditure in the Palestinian population was 5000 NIS (One NIS = 0.28 US Dollars) (PCBS, 2021a) and Deep Line Poverty was 2000 NIS (PCBS, 2021b). Using these data, we considered monthly income of less than 2000 NIS as low monthly income, between 2000 and 5000 NIS as average monthly income, and more than 5000 NIS as high monthly income.

In Table 1, knowledge and concerns about the lockdown section included questions about the type of lockdown, the duration of lockdown, the source of information about the pandemic and lockdown measures, the adequacy of information. Other five questions aimed to assess lockdown understanding, which reflects the knowledge and information the person has about the pandemic and lockdown (Table 2). It was initially evaluated through five statements: (1) lockdown is needed where I live; (2) not committing to lockdown measures will raise the number of cases; (3) measures taken by the government are necessary; (4) lockdown should not only be limited to infected people and those who are in contact with them; and (5) hygiene measures in the house are part of lockdown. A 5-point Likert scale [strongly agree (4), agree (3), neutral (2), disagree (1), and totally disagree (0)] was used to respond to each statement. By summing the points of each statement, a scale from 0 to 20 was created for each respondent. We then used the median as the cutoff point to categorize this outcome into a low level (0–17) and a high level (18–20).

**TABLE 1:** Bivariate analysis of socio-demographic characteristics with anxiety and stress severity (*P*-value presented was Chi-square significance; *N* = 2819).

Variables	*N* (%)	Anxiety severity			*P*-value	Stress severity			*P*-value
		Normal *n* = 2110	Mild to moderate *n* = 566	Severe to extremely severe *n* = 143		Normal *n* = 1726	Mild to moderate *n* = 626	Severe to extremely severe *n* = 467	
**Age**	2819 (100)	Mean = 30.06 *SD* = 11.18	Mean = 27.86 *SD* = 10.27	Mean = 27.10 *SD* = 9.60	<0.001* (ANOVA-test)	Mean = 31.15 *SD* = 11.77	Mean = 27.34 *SD* = 9.33	Mean = 26.10 *SD* = 8.38	<0.001* (ANOVA-test)
**Sex**									
Male	768 (27.2)	619 (29.3)	112 (19.8)	37 (25.9)	<0.001*	549 (31.8)	140 (22.4)	79 (16.9)	<0.001*
Female	2051 (72.8)	1491 (70.7)	454 (80.2)	106 (74.1)		1177 (68.2)	486 (77.6)	388 (83.1)	
**Social status**									
Single	1449 (51.4)	1040 (49.3)	325 (57.4)	84 (58.7)	0.001*	822 (47.6)	344 (55)	283 (60.6)	<0.001*
Relationship	1370 (48.6)	1070 (50.7)	241 (42.6)	59 (41.3)		904 (52.4)	282 (45)	184 (39.4)	
**Residency**									
Village	1380 (49)	1047 (49.6)	275 (48.6)	58 (40.6)	0.184	865 (50.1)	306 (48.9)	209 (44.8)	0.08
City	1292 (45.8)	958 (45.5)	261 (46.1)	73 (51)		780 (45.2)	289 (46.2)	223 (47.8)	
Camp	147 (5.2)	105 (5)	30 (5.3)	12 (8.4)		81 (4.7)	31 (5)	35 (7.5)	
**Geographic area**									
West Bank	2354 (83.5)	1778 (84.3)	465 (82.2)	111 (77.6)	0.066	1464 (84.8)	518 (82.7)	372 (79.7)	0.011*
Gaza strip	270 (9.6)	189 (9)	58 (10.2)	23 (16.1)		149 (8.6)	57 (9.1)	64 (13.7)	
Jerusalem	195 (6.9)	143 (6.8)	43 (7.6)	9 (6.3)		113(6.5)	51 (8.1)	31 (6.6)	
**Educational level**									
Secondary or less	326 (11.6)	241 (11.4)	69 (12.2)	16 (11.2)	0.127	206 (11.9)	73 (11.7)	47 (10.1)	0.018*
College	2211 (78.4)	1647 (78.1)	456 (80.6)	108 (75.5)		1323 (76.7)	506 (80.8)	382 (81.8)	
Master or doctorate	282 (10)	222 (10.5)	41 (7.2)	19 (13.3)		197 (11.4)	47 (7.5)	38 (8.1)	
**Health care worker**									
Yes	332 (11.8)	251 (11.9)	62 (11)	19 (13.3)	0.701	1534 (88.9)	555 (88.7)	398 (85.2)	0.088
No	2487 (88.2)	1859 (88.1)	504 (89)	124 (86.7)		192 (11.1)	71 (11.3)	69 (14.8)	
Monthly income (NIS)*									
<2000	568 (20.1)	399 (18.9)	126 (22.3)	43 (30.1)	0.011*	303 (17.6)	145 (23.2)	120 (25.7)	<0.001*
2000–5000	1552 (55.1)	1177 (55.8)	309 (54.6)	66 (46.1)		970 (56.2)	349 (55.7)	223 (49.9)	
>5000	699 (24.8)	534 (25.3)	131 (23.1)	34 (23.8)		453 (26.2)	132 (21.1)	114 (24.4)	
**Smoking/Shisha**									
Yes	693 (24.6)	511 (24.2)	143 (25.3)	39 (27.3)	0.653	415 (24)	156 (24.9)	122 (26.1)	0.635
No	2126 (75.4)	1599 (75.8)	423 (74.7)	104 (72.7)		1311 (76)	470 (75.1)	345 (73.9)	
**Cohabitating with someone in a high-risk group**									
Yes	1283 (45.5)	911 (43.2)	292 (51.6)	80 (55.9)	<0.001*	747 (43.3)	291 (46.5)	245 (52.5)	0.002*
No	1536 (54.5)	1199 (56.8)	274 (48.4)	63 (44.1)		979 (56.7)	335 (53.5)	222 (47.5)	

*One NIS = 0.28 US Dollars.

**TABLE 2:** Bivariate analysis of lockdown characteristics with anxiety and stress severity (*P*-value presented was Chi-square significance; *N* = 2819).

Variables	*N* (%)	Anxiety severity			*P*-value	Stress severity			*P*-value
		Normal *n* = 2110	Mild to moderate *n* = 566	Severe to extremely severe *n* = 143		Normal *n* = 1726	Mild to moderate *n* = 626	Severe to extremely severe *n* = 467	
**Do you agree that lockdown is important?**									
Yes	2763 (98)	2069 (98.1)	553 (97.7)	141 (98.6)	0.758	1693 (98.1)	612 (97.8)	458 (98.1)	0.879
No	56 (2)	41 (1.9)	13 (2.3)	2 (1.4)		33 (1.9)	14 (2.2)	9 (1.9)	
**Type of lockdown**									
I have to work outside the home	421 (14.9)	309 (14.6)	79(14)	33 (23.1)	0.018*	269 (15.6)	87 (13.9)	65 (13.9)	0.476
Obliged to stay at home	2398 (85.1)	1801 (85.4)	487(86)	110 (76.9)		1457 (84.4)	539 (86.1)	402 (86.1)	
**Any relative or close contact confirmed as COVID-19 positive?**									
Yes	85 (3)	54 (2.6)	23 (4.1)	8 (5.6)	0.032*	44 (2.5)	25 (4)	16 (3.4)	0.165
No	2734 (97)	2056 (97.4)	543 (95.9)	135 (94.4)		1682 (97.5)	601 (96)	451 (96.6)	
**Fear of getting or transmitting COVID-19**									
Yes	2173 (77.1)	1582 (75)	478 (84.5)	113 (79)	<0.001*	1284 (74.4)	519 (82.9)	370 (79.2)	<0.001*
No	646 (22.9)	528 (25)	88 (15.5)	30 (21)		442 (25.6)	107 (82.9)	97 (20.8)	
**Adequately informed about lockdown**									
Yes	2262 (80.2)	1722 (81.6)	432 (76.3)	108 (75.5)	0.007*	1424 (82.5)	487 (77.8)	351 (75.2)	<0.001*
No	557 (19.8)	388 (18.4)	134 (23.7)	35 (24.5)		302 (17.5)	139 (22.2)	116 (24.8)	
**Source of information**									
Television or radio	525 (18.6)	392 (18.6)	102 (18)	31 (21.7)	0.038*	354 (20.5)	101 (16.1)	70 (15)	0.001*
Official government agencies	359 (12.7)	274 (13)	71 (12.5)	14 (9.8)		232 (13.4)	66 (10.5)	61 (13.1)	
A health care worker	159 (5.6)	124 (5.9)	21 (3.7)	14 (9.8)		102 (5.9)	30 (4.8)	27 (5.8)	
Social media	1676 (59.5)	1255 (59.5)	344 (60.8)	77 (53.8)		991 (57.4)	401 (64.1)	284 (60.8)	
Conversation with other people	100 (3.6)	65 (3.1)	28 (4.9)	7 (4.9)		47 (2.7)	28 (4.5)	25 (5.4)	
**Enough food supply to withstand lockdown period**									
Yes	1994 (70.7)	1539 (7.2.9)	371 (65.5)	84 (58.7)	<0.001*	1287 (74.6)	417 (66.6)	290 (62.1)	<0.001*
No	825 (29.3)	571 (27.1)	195 (34.5)	59 (41.3)		439 (25.4)	209 (33.4)	177 (37.9)	
**Lockdown duration**									
1–2 weeks	187 (6.6)	148 (7)	27 (4.8)	12 (8.4)	0.179	119 (6.9)	38 (6.1)	30 (6.4)	0.262
2–3 weeks	847 (30.1)	650 (30.8)	154 (27.2)	43 (30.1)		541 (31.3)	182 (29.1)	124 (26.6)	
3–4 weeks	786 (27.9)	578 (27.4)	172 (30.4)	36 (25.2)		474 (27.5)	167 (26.7)	145 (31)	
>4 weeks	999 (35.4)	734 (34.8)	213 (37.6)	52 (36.4)		592 (34.3)	239 (38.2)	168 (36)	
**Average hours outside home before lockdown**									
<2 h	584 (20.7)	430 (20.4)	121 (21.4)	33 (23.1)	0.399	341 (19.8)	144 (23)	99 (21.2)	0.200
2–6 h	776 (27.5)	596 (28.2)	140 (24.7)	40 (28)		483 (28)	181 (28.9)	112 (24)	
7–10 h	1075 (38.2)	808 (38.3)	221 (39)	46 (32.2)		672 (38.9)	216 (34.5)	187 (40)	
>10 h	384 (13.6)	276 (13.1)	84 (14.8)	24 (16.8)		230 (13.3)	85 (13.6)	69 (14.8)	
**Staying home adherence**									
Low level	1144 (40.6)	946 (44.8)	263 (46.5)	74 (51.7)	0.242	792 (45.9)	291 (46.5)	200 (42.8)	0.428
High level	1675 (59.4)	1164 (55.2)	303 (53.5)	69 (48.3)		934 (54.1)	335 (53.5)	267 (57.2)	
**In-home precautions adherence**									
Low level	1261 (44.7)	842 (39.9)	229 (40.5)	73 (51)	0.032*	714 (41.4)	252 (40.3)	178 (38.1)	0.439
High level	1558 (55.3)	1268 (60.1)	337 (59.5)	70 (49)1		1012 (58.6)	374 (59.7)	289 (61.9)	
**Lockdown understanding**									
Low level	1283 (45.5)	946 (44.8)	250 (44.2)	65 (45.5)	0.946	774 (44.8)	284 (45.4)	203 (43.5)	0.814
High level	1536 (54.5)	1164 (55.2)	316 (55.8)	78 (54.5)		952 (55.2)	342 (54.6)	264 (56.5)	
**Self-rating of lockdown commitment**	2819 (100)	Mean = 8.49 *SD* = 1.81	Mean = 8.60 *SD* = 1.88	Mean = 8.15 *SD* = 2.11	0.034* (ANOVA-test)	Mean = 8.45 *SD* = 1.84	Mean = 8.52 SD = 1.81	Mean = 8.63 *SD* = 1.87	0.149 (ANOVA-test)

*Indicates significant P-value.

Precautionary measures against COVID-19 section included 5 questions to assess staying home adherence, and five questions to assess in-home precautions adherence. Staying home adherence reflects the compliance of the individual to the main instruction given by the government: “Do not leave the house if it is not necessary” (Table 2). It was initially evaluated through five statements: (1) going grocery shopping or to the bakery; (2) going out meeting friends or family; (3) going out to spend time and have fun; (4) attending social events; and (5) going to the pharmacy. The answer to each statement was composed of [never going out (3), some days (2), more than half of days (1), and every day (0)].

In-home precautions adherence reflects the compliance to infection control measures while staying inside the home to minimize the spread of infection between family members. It was initially evaluated through five statements: (1) washing hands for 20 s or more; (2) decrease the time of interaction with other family members; (3) washing hands after returning from outside; (4) sneezing appropriately according to guidelines (using a tissue or using elbow); and (5) not sharing towels and items between family members. The answer to each statement was composed of [never do them (0), do them sometimes (1), do them most of the time (2), and always do them (3)].

For these last two outcomes separately, we summed up the points of each statement. A scale from 0 to 15 was created for each respondent. Then the median was used as the cutoff point to categorize staying home adherence outcome to a low level (0–12) and a high level (13–15) while categorizing in-home precautions adherence outcome to a low level (0–10) and a high level (11–15).

In the Depression Anxiety Stress Scale (DASS) section, we used the Arabic form of the DASS-21 scale. It is an instrument that included 42-self-report items designed to measure the three related negative emotional states of depression, anxiety, and tension/stress. A short version, the DASS-21, is available with 7 items per scale (UNSW, 2021). The DASS-21 scale showed excellent Cronbach’s alpha values of 0.81, 0.89, and 0.78 for the subscales of depressive, anxiety, and stress respectively (Coker et al., 2018). In this study, Cronbach alpha was 0.82 (anxiety), and 0.89 (stress) which indicating high consistency for the relevant psychometric scales.

The results of DASS-21 supported the universality of depression, anxiety, and stress across cultures (Moussa et al., 2017). The DASS-21 scale was developed from the original DASS-42, which was invented by Sydney H. Lovibond and Peter F. C. Lovibond (1995) (Lu et al., 2018). It has been widely used since its development and showed good psychometric properties (factorial validity and reliability), so it can be used as a reliable and valid instrument for measuring depression, anxiety, and stress symptoms (Antony et al., 1998; Vasconcelos-Raposo et al., 2013; Beaufort et al., 2017; Le et al., 2017; Lu et al., 2018).

The anxiety subscale of DASS-21 assesses autonomic arousal, skeletal muscle association, situational anxiety, and subjective experience of anxious affect. The Stress scale assesses difficulty relaxing, nervous arousal, and being easily upset/agitated, irritable/over-reactive, and impatient. Subjects were asked to use 4-point severity/frequency scales to rate the extent to which they have experienced each state over the past week. As in:

0 – Did not apply to me at all.
1 – Applied to me to some degree, or some of the time.
2 – Applied to me to a considerable degree or a good part of the time.
3 – Applied to me very much or most of the time.

Anxiety and stress scores were calculated by summing the scores for the relevant items. The scores on the DASS-21 were multiplied by 2 to calculate the final score. Scores are shown as the following (Xiang et al., 2020):

Anxiety scores: Normal (0–7), mild (8–9), moderate (10–14), severe (15–19), and extremely severe (20+). Stress scores: Normal (0–14), mild (15–18), moderate (19–25), severe (26–33), and extremely severe (34+). DASS-21 scores may be presented in five categorical levels. However, in this study, and according to a standardized cut-offs, we merged mild with moderate and severe with extremely severe cut-off scores to facilitate the multivariate analysis, as some cells showed less than 5 cases in some categorical independent variables and this is usually acceptable (Allabadi et al., 2019).

### Statistical Analysis

Data were entered into the 27th version of IBM SPSS Software (IBM SPSS Statistics for Windows, Version 27.0. Armonk, NY, United States: IBM Corp). We conducted the descriptive analysis (median, mean, and standard deviation) for continuous variables and (frequencies/percentages) for categorical independent variables. Independent *t*-test was used to test for significance among continuous variables and Chi-square test for categorical variables. Variables showed to be significant in bivariate analysis (with *P*-value less than 0.05) were included in the multinomial logistic regression models to predict the factors associated with each anxiety and stress severity degrees and presented as odds ratio and 95% CI.

## Results

### Characteristics of the Study Population

In this study, 2819 individuals completed and returned the questionnaire (Table 1). The mean (range) age of respondents was 29.47 (18–71) years with SD of 10.97 years. More than two thirds (72.6%) of respondents were females. Almost half of them (51.4%) were single. The majority lived in the West Bank (83.5%) and only 9.6% in Gaza. Around 55.1% had an average monthly income of 2000-5000 New Israeli Shekels per month (One NIS = 0.28 US Dollars).

Most of the participants (78.4%) were college students or recent graduates. Another 10% were masters or doctoral students. Almost one quarter (24.6%) were smokers and only 11.8% were health care workers. About 45.5% reported cohabitation with someone who is of a high-risk group. Results showed that 59.4, 55.3, and 54.5% of respondents reported high levels of staying home adherence, in-home precautions adherence, and lockdown understanding; respectively.

### Lockdown Characteristics of the Population

As shown in Table 2,98% of respondents believed that lockdown is important, and 77.1% expressed fear of getting COVID-19 or transmitting it to others. Only 14.9% had jobs that required them to go outdoors, and only 3% had at least one relative with confirmed COVID-19. The two most common sources of information about the lockdown measures were social media and television or radio (59.5 and 18.6%, respectively). Nearly, 80.2% considered themselves as properly informed about the lockdown. In addition, 29.3% self-reported inadequate food supply to withstand the lockdown period.

Duration in lockdown at the time the participants filled the survey ranged from less than 2 weeks in 6.6% to more than 4 weeks in 35.4% of the participants. Most people (38.2%) used to spend between 6 and 10 h outside the home before lockdown, 20.7% spent less than 2 h and only 13.6% spent more than 10 h (see Table 2 for more details).

### Prevalence of Anxiety and Stress in a Bivariate Analysis

The prevalence of anxiety was 25.15% (*n* = 709; 20.08% with mild/moderate and 5.07% with severe/extremely severe). The prevalence of stress was 38.77% (*n* = 1093; 22.21% with mild/moderate and 16.56% with severe/extremely severe).

**FIGURE 1:**
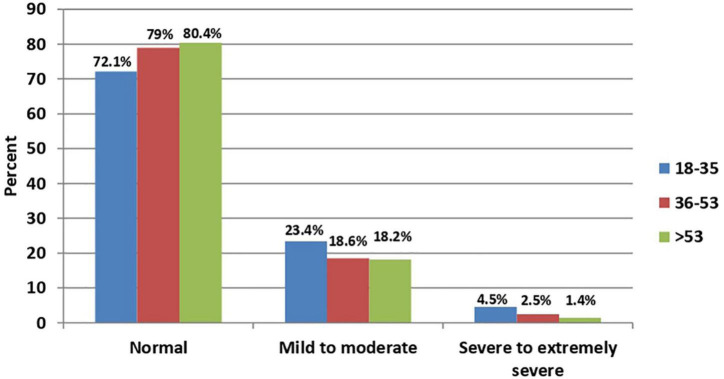
Age distribution among anxiety severity (*P*-value < 0.001; *N* = 2,819).

**FIGURE 2:**
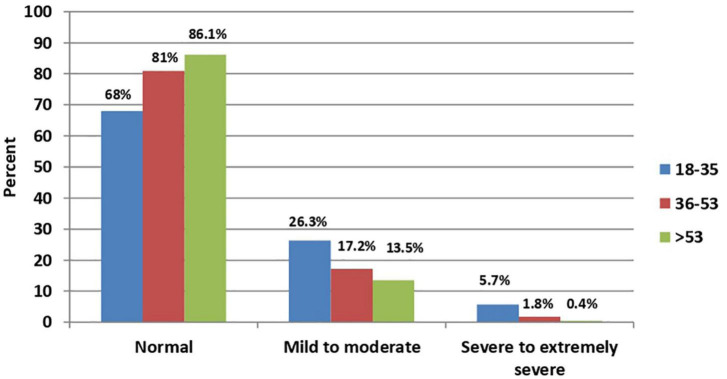
Age distribution among stress severity (*P*-value < 0.001; *N* = 2,819).

**FIGURE 3:**
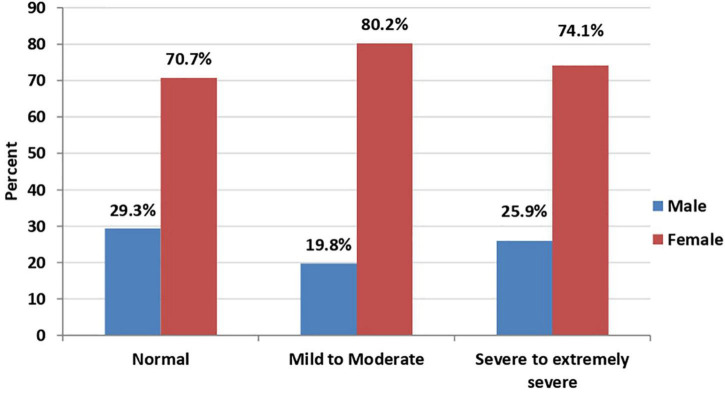
Sex distribution among anxiety severity (*P*-value < 0.001; *N* = 2,819).

**FIGURE 4:**
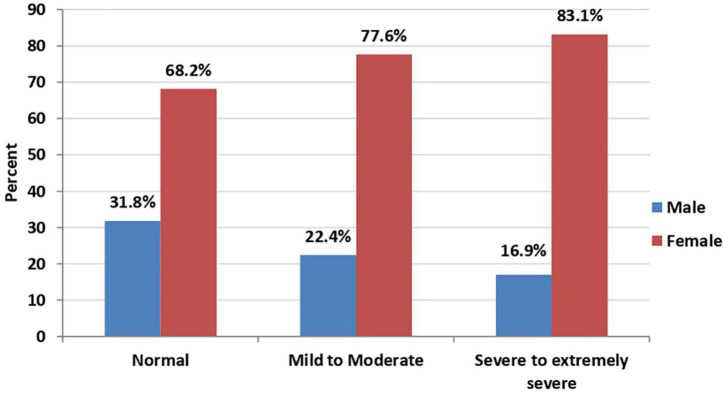
Sex distribution among stress severity (*P*-value < 0.001; *N* = 2,819).

In bivariate analysis, a statistically significant association was found between age (Figures 1, 2), sex (Figures 3, 4), social status, monthly income, and cohabitation with someone who was at high-risk group with both anxiety and stress severity (*p*-value < 0.05; Table 1). Geographic area and educational level were also found to be statistically significant with stress severity, but not with anxiety.

On the other hand, the type of lockdown, having relatives or acquaintances infected, and the in-home precautions adherence were statistically significant with anxiety but not stress. Fear of getting or transmitting COVID-19, proper information about lockdown, the source of information, and enough food supply to withstand the lockdown were significant in both anxiety and stress (*p*-value < 0.05; Table 2).

The fear of transmission had strong associations with higher levels of anxiety and stress severities (*p*-value < 0.001, Table 2). People who complained of inadequate food supply to withstand the lockdown period made the majority in all categories of both anxiety and stress (*p*-value < 0.001, Table 2). High-level commitment represented the majorities in the normal and the mild to moderate categories in the anxiety severity with 60.1 and 59.5%, respectively (Table 2).

### Multinomial Analysis of Anxiety Severity Predictors

A multinomial regression model for the variables associated with anxiety severity is shown in Table 3. As shown, cohabitation with someone at high-risk group was significantly predictive of anxiety severity (mild/moderate [OR (95%CI) = 0.72 (0.60–0.87)] and severe/extremely severe degrees [OR (95%CI) = 0.62 (0.44–0.88)]). People who reported knowing or being in contact with any confirmed case of COVID-19 personally were significantly more likely to have higher anxiety degree compared to people who reported not knowing or being in contact with confirmed cases (mild/moderate [OR (95%CI) = 0.57 (0.34–0.94)] and severe/extremely severe degrees [OR (95%CI) = 0.41 (0.19–0.90)]).

**TABLE 3:** Multinomial regression model for the variables associated with anxiety severity^#^ (*N* = 2819).

Variable	Mild to moderate				Severe to extremely severe			
	*B*	*SE*	OR (95%CI)	*P*-value	*B*	*SE*	OR (95%CI)	*P*-value
**Age** (continuous)	–0.11	0.006	0.99 (0.98–1.00)	0.070	–0.02	0.012	0.98(0.96–1.00)	0.056
**Sex**								
Male	–0.75	0.126	0.57 (0.44–0.72)	< 0.001*	–0.40	0.215	0.67 (0.44–1.02)	0.061
Female*	−	−	−	−	−	−	−	−
**Social status**								
Single	0.23	0.126	1.25 (0.98–1.61)	0.073	–0.12	0.229	1.13 (0.72–1.77)	0.605
Relationship*	−	−	−	−	−	−	−	−
**Monthly income (NIS)**								
< 2000	0.05	0.151	1.05 (0.78–1.41)	0.749	0.21	0.256	1.23 (0.74–2.03)	0.421
2000–5000	0.01	0.121	1.01 (0.80–1.28)	0.929	–0.23	0.224	0.80 (0.51–1.23)	0.307
> 5000*	−	−	−	−	−	−	−	−
**Cohabitation with someone in a high-risk group**								
No	–0.33	0.097	0.72 (0.60–0.87)	0.001*	–0.48	0.178	0.62 (0.44–0.88)	0.007*
Yes*	−	−	−	−	−	−	−	−
**Type of lockdown**								
I have to work outside the home	0.02	0.145	1.02 (0.77–1.35)	0.905	0.48	0.224	1.62 (1.04–2.51)	0.033*
Obliged to stay at home*	−	−	−	−	−	−	−	−
**Any relative or close contact confirmed as COVID-19 positive?**								
No	–0.57	0.260	0.57 (0.34–0.94)	0.029*	–0.89	0.399	0.41 (0.19–0.90)	0.026*
Yes*	−	−	−	−	−	−	−	−
**Fear of getting or transmitting COVID-19**								
No	–0.61	0.130	0.55 (0.42–0.70)	< 0.001*	–0.31	0.219	0.73 (0.48–1.13)	0.155
Yes*	−	−	−	−	−	−	−	−
**Adequately informed about lockdown**								
No	0.30	0.120	1.35 (1.06–1.71)	0.013*	0.20	0.214	1.22 (0.80–1.85)	0.361
Yes*	−	−	−	−	−	−	−	−
**Source of information**								
Television or radio	–0.38	0.263	0.68 (0.41–1.14)	0.148	0.12	0.459	1.13 (0.46–2.77)	0.795
Official government agencies	–0.43	0.274	0.65 (0.38–1.11)	0.117	–0.41	0.503	0.66 (0.25–1.78)	0.414
A health care worker	–0.93	0.336	0.39 (0.20–0.76)	0.006*	0.22	0.505	1.25 (0.47–3.36)	0.659
Social media	–0.47	0.243	0.63 (0.39–1.01)	0.054	0.35	0.430	0.71 (0.30–1.64)	0.416
Conversation with other people*	−	−	−	−	−	−	−	−
**Enough food supply to withstand lockdown period**								
No	0.42	0.109	1.52 (1.23–1.88)	< 0.001*	0.63	0.191	1.88 (1.30–2.74)	0.001*
Yes*	−	−	−	−	−	−	−	−
**In-home precautions adherence**								
Low level	–0.03	0.099	0.97 (0.80–1.18)	0.761	0.00	0.180	1.00 (0.70–1.42)	0.998
High level*	−	−	−	−	−	−	−	−
**Self-rating lockdown commitment** (continuous)	0.12	0.030	1.01 (0.96-1.07)	0.680	–0.07	0.047	0.94 (0.86-1.03)	0.159

^#^Reference category: Normal; *Reference category (One NIS = 0.28 US Dollars).

*OR, odds ratio; CI, confidence interval; SE, standard error.*

*Likelihood Ratio Test of the final model fitting significance was < 0.001. Pearson chi-square test for model goodness-of-fit significance was 0.30.*

Those who reported inadequate food supply during lockdown were more likely to have a higher degree of anxiety (mild/moderate [OR (95%CI) = 1.52 (1.23–1.88)] and severe/extremely severe degrees [OR (95%CI) = 1.88 (1.30–2.74)]).

Males were significantly less likely to have mild/moderate anxiety compared to females [OR (95%CI) = 0.057 (0.44–0.72)]. Those with essential jobs (that required leaving home even during lockdown) were significantly more likely to have severe/extremely severe anxiety compared to those who were asked by the government to stay at home [OR (95%CI) = 1.62 (1.04–2.51)]. Moreover, having adequate information about lockdown measures and no fear of being infected were significantly protective against anxiety (see Table 3).

It should be noted that age, social status, monthly income, in-home precautions adherence level, self-rating of commitment, and source of information did not remain significant after adjusting for other variables in the multinomial regression model.

### Multinomial Analysis of Stress Severity Predictors

Multinomial regression model for the variables associated with stress severity is shown in Table 4. As shown, age was inversely associated with stress severity (mild/moderate degree [OR (95%CI) = 0.97 (0.95–0.98)] and severe/extremely severe [OR (95%CI) = 0.96 (0.94–0.97)]). Males were less likely to report either mild/moderate or severe/extremely severe degrees of stress compared to females ([OR (95%CI) = 0.64 (0.51–0.81)], [OR (95%CI) = 0.40 (0.30–0.52)], respectively).

**TABLE 4:** Multinomial regression model for the variables associated with stress severity^#^ (*N* = 2819).

Variable	Mild to moderate				Severe to extremely severe			
	*B*	*SE*	OR (95%CI)	*P*-value	*B*	*SE*	OR (95%CI)	*P*-value
**Age** (continuous)	–0.04	0.006	0.97 (0.95–0.98)	< 0.001*	–0.05	0.008	0.96(0.94–0.97)	< 0.001*
**Sex**								
Male	–0.44	0.116	0.64 (0.51–0.81)	< 0.001*	–0.93	0.143	0.40 (0.30–0.52)	< 0.001*
Female*	−	−	−	−	−	−	−	−
**Social status**								
Single	–0.11	0.125	0.90 (0.70–1.15)	0.394	–0.02	0.142	0.98 (0.74–1.30)	0.901
Relationship*	−	−	−	−	−	−	−	−
**Geographic area**								
West Bank	–0.29	0.185	0.75 (0.52–1.08)	0.121	0.00	0.222	1.00 (0.65–1.55)	0.999
Gaza	–0.57	0.245	0.56 (0.35–0.91)	0.019*	0.09	0.271	1.09 (0.64–1.86)	0.749
Jerusalem*	−	−	−	−	−	−	−	−
**Educational level**								
Secondary or less	0.08	0.223	1.09 (0.70–1.68)	0.711	–0.22	0.256	0.80 (0.49–1.32)	0.387
College	0.10	0.180	1.11 (0.78–1.57)	0.578	–0.13	0.201	0.88 (0.59–1.31)	0.527
Master or doctorate*	−	−	−	−	−	−	−	−
**Monthly income (NIS)**								
< 2000	0.34	0.157	1.41 (1.03–1.91)	0.030*	0.07	0.170	1.08 (0.77–1.50)	0.661
2000–5000	0.18	0.123	1.19 (0.94–1.52)	0.156	–0.16	0.136	0.86 (0.66–1.12)	0.254
> 5000*	−	−	−	−	−	−	−	−
**Cohabitation with someone in a high-risk group**								
No	–0.14	0.097	0.87 (0.72–1.06)	0.164	–0.41	0.109	0.67 (0.54–0.82)	< 0.001*
Yes*	−	−	−	−	−	−	−	−
**Fear of getting or transmitting COVID-19**								
No	–0.53	0.124	0.59 (0.46–0.75)	< 0.001*	–0.26	0.133	0.77 (0.60–1.01)	0.055
Yes*	−	−	−	−	−	−	−	−
**Adequately informed about lockdown**								
No	0.21	0.121	1.24 (0.98–1.57)	0.079	0.33	0.133	1.39 (1.07–1.80)	0.014*
Yes*	−	−	−	−	−	−	−	−
**Source of information**								
Television or radio	–0.59	0.275	0.56 (0.32–0.95)	0.033*	–0.66	0.295	0.52 (0.29–0.92)	0.025*
Official government agencies	–0.76	0.286	0.51 (0.29–0.90)	0.020*	–0.44	0.301	0.64 (0.36–1.16)	0.141
A health care worker	–0.66	0.326	0.52 (0.27–0.98)	0.042*	–0.62	0.344	0.54 (0.28–1.06)	0.074
Social media	–0.39	0.255	0.68 (0.41–1.12)	0.131	–0.52	0.270	0.59 (0.35–1.01)	0.052
Conversation with other people*	−	−	−	−	−	−	−	−
**Enough food supply to withstand lockdown period**								
No	0.40	0.110	1.49 (1.20–1.85)	< 0.001*	0.67	0.122	1.96 (1.54–2.49)	< 0.001*
Yes*	−	−	−	−	−	−	−	−

^#^Reference category: Normal; *Reference category (One NIS = 0.28 US Dollars).

*OR, odds ratio; CI, confidence interval; SE, standard error.*

*Likelihood Ratio Test of the final model fitting significance was < 0.001. Pearson chi-square test for model goodness-of-fit significance was 0.397.*

Those who reported inadequate food supply during lockdown were more likely to have a higher degree of stress compared to those who did not (mild/moderate degree [OR (95%CI) = 1.49 (1.20–1.85)] and severe/extremely severe [OR (95%CI) = 1.96 (1.54–2.49)]). Cohabitation with someone at the high-risk group was a significant predictor of severe/extremely severe stress degree [OR (95%CI) = 0.67 (0.54–0.82)]. Having adequate information about lockdown measures and no fear of being infected were significantly protective against stress (see Table 4).

Those with a low monthly income (less than 2000 New Israeli Shekels) were significantly more likely to have mild/moderate stress compared to average and high monthly incomes [OR (95%CI) = 1.41 (1.03–1.91)]. Those who reported television or radio as a source of information were significantly less likely to have higher stress degree compared to people who depend on a conversation with other people as source of information (mild/moderate degree [OR (95%CI) = 0.56 (0.32–0.95)] and severe/extremely severe [OR (95%CI) = 0.52 (0.29–0.92)]).

Finally, residing in Gaza was found to be significantly protective against stress severity compared to the West Bank and Jerusalem. It should be noted that social status and educational level did not remain significant after adjusting for other variables in the multinomial regression model.

## Discussion

In this study, the prevalence of anxiety and stress among the Palestinian general population during the COVID-19 pandemic lockdown were found to be 25.15 and 38.77%; respectively. It is worth noting that 20.3% of our population was found to have both anxiety and stress, and only 4.2% have a severe/extremely severe degree of both anxiety and stress. Comparable data concerning the Palestinian population prior to the COVID-19 lockdown are not readily available. The best available data come from a previous study conducted 13 years ago in the West Bank and Gaza. This study reported a prevalence of anxiety of 16.3% and acute stress of 8.3% in adults (Espié et al., 2009).

At the global level, a recent study in Italy used DASS-21 scale. It reported a prevalence of 18.7% of anxiety and 27.2% of stress in the Italian general population during COVID-19 pandemic (Mazza et al., 2020). Another study in Northern Spain found that the anxiety rate was 26.02% and the stress rate was 33.5% in the Spanish population during the same period (Ozamiz-Etxebarria et al., 2020). In United Kingdom, it was found that the prevalence of anxiety was 21.63% during the COVID-19 pandemic (Shevlin et al., 2020). In China, a study used the DASS-21 scale and found a prevalence of 37.4% of anxiety and 32.1% of stress (Wang et al., 2020). Our findings regarding anxiety and stress prevalence in the Palestinian population are comparable to those found in other populations during COVID-19 pandemic. Stress and anxiety during the pandemic came from the uncertainty and wide-range of expectations of the future rather than infection rate (Palestine had a low number of COVID-19 cases and death at the time of the survey in comparison to these countries).

Inadequate food supply was found to be the only factor that positively associated with all degrees of both anxiety and stress. In our sample, 51% of respondents who reported inadequate food supply were also from low monthly income group, so whether inadequacy in food supply was a pre-existing condition or triggered recently by lockdown measures is unknown by our cross-sectional study and needs further investigation. However, we do believe that low socio-economic status had affected the adequacy of food supply as these two factors are cross related at all levels mainly the economical and therefore the ability to afford food in the families. This is indeed could be supported by the finding that, people with low monthly income was found to be more likely to have a mild/moderate degree of stress compared to people with a high monthly income [OR (95%CI) = 1.41 (1.03–1.91)]. Further mechanism behind that could be explained by that many jobs were lost due to social distancing and lockdown measures, which added more to the financial loss of individuals and society. In United Kingdom, Canada, and Korea, it was found that lost income and people with low monthly income were more likely to have anxiety and stress than people with a high monthly income during the same pandemic, during SARS 2003 and during MERS 2015 (Hawryluck et al., 2004; Jeong et al., 2016; Shevlin et al., 2020). A study in Bangladesh found a greater prevalence of sleep disturbance among participants who or anyone from their family members lost jobs during this pandemic, as losing jobs created massive insecurity of meeting livelihoods (Ara et al., 2020). A systematic review of different studies from 39 countries reported an estimated prevalence of sleep problems of 31% among healthcare professionals, 18% among the general population, and 57% among COVID-19 patients (Alimoradi et al., 2021). The most possible explanation and mechanism of this relationship may be explained by the economic stress disseminated all over the countries during lockdown where low monthly income group would be the most vulnerable. It is worth mentioning that Palestine is classified as a middle low-income country and has a low socio-economic status (World Bank, 2021). However, a study on six Arabic countries nearby Palestine found that no differences were noted in rates of anxiety or stress between low-income countries and high-income countries (Al Omari et al., 2020).

Age showed an inverse relationship with stress severity, but not anxiety. A systematic review of 43 large studies concluded that anxiety was consistently associated with younger age (Santabárbara et al., 2021b). Two studies in Spain (González-Sanguino et al., 2020) and Northern Spain (Ozamiz-Etxebarria et al., 2020) found that the younger participants were more likely to have both stress and anxiety. An Italian study concluded that age and anxiety, but not stress, had an inverse relationship (Mazza et al., 2020). However, a Chinese study found that age was not associated with anxiety or stress level (Wang et al., 2020). All of these studies were held during the same pandemic and used the DASS-21 scale. According to the Palestinian Central Bureau of Statistics (PCBS), 66.3% of the Palestinians in 2020 were younger than 29 years old, so Palestinian society has a very young age structure, as reflected in this study (PCBS, 2021a). As the vast majority of the Palestinian population is young they could be more vulnerable to stress and anxiety due to lockdown measures. Usually young population spend more time outdoor and this lockdown could have significant impact on their psychological health. Moreover, during the lockdown, college and university students shifted to an online learning platforms, this might have been associated with increased stress and anxiety (UNESCO, 2021). Lockdown measures also limited job prospects for the large portion of newly graduated students which might explain the higher levels of stress among younger adults in our study. This is in accordance to some other studies where a systematic review of different studies from four countries showed that fear of COVID-19 was associated with increased future career anxiety and perceived job insecurity (Rajabimajd et al., 2021).

In this study, females were more likely to have mild/moderate degree of anxiety and both mild/moderate and severe/extremely severe degrees of stress compared to males. A systematic review of 43 studies during COVID-19 found a significantly higher anxiety levels in women, this could be explained and justified by the differences in brain chemistry, women are usually caregivers, and thus had a reduced ability to perform their work (Santabárbara et al., 2021b). Similar findings came from United Kingdom regarding anxiety (Shevlin et al., 2020). Other studies during COVID-19 did not find a difference between females and males (Hu et al., 2020). A Chinese study (Wang et al., 2020), an Italian study (Mazza et al., 2020), and an Arabic study (Al Omari et al., 2020) found that females were more likely to have both anxiety and stress during the same pandemic. Another study in Bangladesh found a higher prevalence of sleep disturbance and anxiety among women (Ara et al., 2020). A Jordanian study found that female gender was significantly associated with high level of distress (Khatatbeh et al., 2021). Another possible explanation and mechanism behind this relationship could be that anxiety and stress during the pandemic beat cultural differences, as different types of societies represent the same results. In the Palestinian society at least, there is a patriarchal notion that women’s loyalty mostly lies with their home duties and their families (ReliefWeb, 2020). According to a 2017 study in Palestine, 80% of men and 60% of women believe a woman’s most important role is home-care (ReliefWeb, 2020). Consequently, home confinement may have increased household responsibilities, which may disproportionately affect Palestinian women (ReliefWeb, 2020). A report by UN Women found that 76% of women had lost their income (compared to 65% men) (UN Women, 2021).

Cohabitation with someone at the high-risk group was a significant predictor of both anxiety and stress. This relationship could be due to the fear of losing them, as this condition makes them vulnerable to the more devastating association of the COVID-19 virus. On one hand, a significant association was noticed between cohabitation with someone at the high-risk group and the adherence to in-home precautions in our population, most probably in an attempt to protect them from the virus. The delay in medical supplies including necessary medication to the high-risk group, during the COVID-19 pandemic could also have a role (, 0000) and forcing this particular group to fight against multiple stressors.

People who reported knowing cases confirmed with COVID-19 were more likely to have both mild/moderate and severe/extremely severe degrees of anxiety. This is consistent with other studies from Spain and Italy (Hawryluck et al., 2004; Rodríguez-Rey et al., 2020). People who reported fear of getting COVID-19 or transmitting it was more likely to have a mild/moderate degree of both anxiety and stress compared to people who didn’t report the same feelings. This is also noted in studies conducted on different countries (Barzilay et al., 2020; Lei et al., 2020; Upadhyay et al., 2020). The same was observed in a meta-analysis pooled data from 91 studies with 88 320 participants from 36 countries that showed associations between fear of COVID-19 and mental health-related factors were mostly moderate (Fisher’s *z* was 0.54 for anxiety and 0.42 for stress) (Alimoradi et al., 2022).

Respondents who expressed being adequately informed about lockdown measures were less anxious and distressed compared to those who complained of having inadequate information. This is consistent with other studies (Sim et al., 2010; Wang et al., 2020). Working in jobs that required going outside the home (e.g., health care workers, police officers etc.) was found to be a significant predictive factor of anxiety. Being the frontline fighters, healthcare workers were also more likely to develop anxiety due to fears of becoming infected or transmitting the infection to others (Dubey et al., 2020). The lockdown aimed to decrease interactions between people, to avoid the spread of infection, and to make contact tracing easier as new cases stem outside sources during work and are then transmitted to a household member.

It is worth mentioning that in our study educational level had no impact on anxiety or stress severity in multivariate analysis. Similarly, a Bangladeshi study found no association between educational level and stress or anxiety (Ara et al., 2020). In another Italian study, it was noted that people with higher educational levels were more likely to have stress compared to people with low educational levels (Mazza et al., 2020). Interestingly, an Egyptian study found that university students have a higher degree of stress more than non-educated and highly-educated people (El-Zoghby et al., 2020). In Palestine, this relationship between the educational level and the level of anxiety and stress, could be explained by that an advanced educational level does not guarantee a job. According to the Palestinian Central Bureau of Statistics (PCBS) in 2018, the unemployment rate rose to 58% among graduates aged 20–29 years (PCBS, 2021a). In Palestine, however, most new graduates are entering the workforce for the first time after completing their education (, 0000). Therefore, the educated individuals in the Palestinian society could not have a lot of worries so their level of stress and anxiety might have not been affected by the lockdown as they used to this situation.

This study is limited by the sampling technique, which may have introduced selection bias. Importantly, 78% of participants were females which might over-estimate the stress and anxiety severity and therefore our anxiety and stress rates should be interpreted with caution. Furthermore, due to social distancing during the lockdown, we disseminated the survey on social media and this might in part exclude people who didn’t have access to the internet and social media. Meanwhile, this was the only possible procedure during the lockdown with faster and safer collection of the required information.

This study was a cross-sectional web-based survey and therefore data can’t be used to infer causality because temporality is not known, recall and/or systematic bias were also possible. Furthermore, over and/or under-estimation of some measures might have been occurred. However, it should be noted that this study has several strengths including a large sample size and the sampling timeframe that corresponded to the peak surge of COVID-19 cases in Palestine (COVID-19, 2022).

## Conclusion

We reported high rates of anxiety and stress and different predictors of their severities among the Palestinian general population during COVID-19 lockdown. Certain key groups who might be more vulnerable to COVID-19 lockdown measures were identified. Particularly, those with had low socioeconomic status, younger and female.

Implications of these findings include better management of the pandemic and alternative approaches to address the socioeconomic inequalities, given their impact on the psychological health of the population. This involves changing polices, providing alternative economical sources for those who are in-need, and spreading more awareness regarding the pandemic. Current and future response-plans need to take into account the psychological burdens of pandemics and how to mitigate them. This is crucial as communities move forward and begin to emerge from COVID-19 crisis. Further research is needed to track whether these vulnerable groups show higher levels of psychological impacts at later stages of the pandemic.

## Data Availability Statement

The original contributions presented in this study are included in the article/supplementary material, further inquiries can be directed to the corresponding author.

## Ethics Statement

The studies involving human participants were reviewed and approved by An-Najah National University IRB Committee. Written informed consent for participation was not required for this study in accordance with the national legislation and the institutional requirements.

## Author Contributions

HA, MH-Y, NY, and TA designed study protocol and drafted the manuscript. HA coordinated the study protocol and conducted the statistical analysis. MH-Y, NY, and TA collected the data. All authors read and approved the final manuscript.

## Conflict of Interest

The authors declare that the research was conducted in the absence of any commercial or financial relationships that could be construed as a potential conflict of interest.

## Publisher’s Note

All claims expressed in this article are solely those of the authors and do not necessarily represent those of their affiliated organizations, or those of the publisher, the editors and the reviewers. Any product that may be evaluated in this article, or claim that may be made by its manufacturer, is not guaranteed or endorsed by the publisher.
